# Active Aging through Volunteerism: A Longitudinal Assessment of Perceived Neighborhood Safety as a Predictor among Older Adults, 2008-2018

**DOI:** 10.1155/2021/5185264

**Published:** 2021-10-27

**Authors:** Erin G. Grinshteyn, Judith A. Sugar

**Affiliations:** ^1^Health Professions Department, School of Nursing and Health Professions, University of San Francisco, 2130 Fulton Street, San Francisco, CA 94177, USA; ^2^School of Public Health/0274, University of Nevada, Reno, 1664 N. Virginia Street, Reno, NV 89557, USA

## Abstract

Volunteering can play an important role in active aging. The resource theory of volunteering posits that volunteerism depends on human, social, and cultural capital. Benefits of volunteering have been documented at the micro-, meso-, and macrolevels, positively affecting individual older people as well as their local communities and society at large. Taking a process-oriented theoretical approach, this study focused on the mesolevel factor of the environment with the purpose of determining the relationship between perceived neighborhood safety and volunteerism over the course of a decade and the extent to which this relationship differs by gender and race. Longitudinal data from the Health and Retirement Study in the United States of America between 2008 and 2018 were used (*N* = 72,319 adults 60 years and older). Generalized estimating equations (GEE) with robust standard errors were employed while controlling for a number of covariates. A third of the sample volunteered in the past year (33%). The probability of volunteering among older adults who rated their perceived neighborhood safety as excellent was greater compared with those who rated their perceived neighborhood safety as fair/poor after controlling for all other model covariates (ME: 0.03, 95% CI: 0.02, 0.05). Among males rating their perceived neighborhood safety as excellent, the probability of volunteering was higher (ME: 0.04, 95% CI: 0.02, 0.07). Among females, the probability of volunteering was higher among those who perceived their neighborhood safety to be excellent (ME: 0.03, 95% CI: 0.01, 0.05) or very good (ME: 0.02, 95% CI: 0.00, 0.04). White respondents who rated their neighborhood safety as excellent (ME: 0.05, 95% CI: 0.03, 0.07) or very good (ME: 0.04, 95% CI: 0.02, 0.06) had a higher probability of volunteerism. Results were not significant among Black respondents and those who described their race as “other.” This study's process-oriented theoretical approach indicates that initiatives aimed at improving neighborhood safety and older adults' perceptions of neighborhood safety could increase social capital and lead older adults to engage in more volunteering, providing benefits at micro-, meso-, and macrolevels—to older individuals, their local communities, and society at large.

## 1. Introduction

Volunteering is an important arena for active aging, and its effects can be seen at micro-, meso-, and macrolevels. At the microlevel, volunteering impacts individuals' physical and mental health; at the mesolevel, volunteering can be supported or diminished by the safety of their physical and social environments; and at the macrolevel, volunteering contributes to economies, communities, and society. Volunteering can take place informally, outside of an organizational context, and formally within the context of organizations, typically public agencies or nonprofit institutions. To get a glimpse of the enormous contributions of older Americans' volunteer activities, note that in 2017, 11 million older Americans volunteered in a Senior Corps program (RSVP, Foster Grandparents, Senior Companions), contributing a total of almost 2 billion hours of service in thousands of communities with an economic value of $454 billion [1].

Previous research has shown that older adults are more likely to participate in volunteer activities when they perceive that the neighborhood in which they live is safe [2]. The goals of this paper are to better understand the relationship between perceived safety and volunteerism over time and to assess this relationship by gender and race, given existing differences in both perceived safety and volunteerism.

### 1.1. Benefits of Volunteerism

The effects of volunteering can be seen at the micro-, meso-, and macrolevels. Among the many benefits of volunteering for older people at the microlevel are increases in their psychological well-being, including life satisfaction, self-perception, happiness, and sense of purpose [3–5], and increases in their physical health, including protective effects on mortality, lower risk of cardiovascular incidents, and fewer functional limitations [6, 7].

At the mesolevel, the benefits of volunteerism are numerous. One study of older adult volunteers found that almost all volunteers (90%) reported that either the people or the community they served were better off as a result of their volunteerism [8]. Volunteerism has also been associated with increased community awareness and reductions in stereotypes [9]. Additional benefits to the community include improved collective efficacy, community resilience, and social cohesion [10–12]. Research has posited that informal volunteering specifically is fundamental to social capital, which makes volunteerism a crucial component of the community [13].

In addition to the evidence that volunteering positively affects older adults' physical and mental health and communities, there is growing recognition of the potential for benefits at the macrolevel—that the human, social, and cultural capital of older adults can be directed to help solve important societal issues. With their accumulated wisdom, skills, and talents, older adults have much they can contribute. Programs are being developed to take advantage of the dividends that longer, healthier lives can bring. Two examples that do just that are the Experience Corps and the Retirees in Service to the Environment (RISE) program. Experience Corps is an evidence-based program designed to leverage the social capital of diverse older adults to improve the academic success of at-risk children in kindergarten through third grade [14]. Teams of older volunteers are trained to work in schools where they help children learn to read, provide social-emotional support, and help teachers with classroom activities. RISE recruits and provides training and educational workshops for older volunteers who then select an environmental project that will improve their community [15]. Projects have included, among others, developing environmental awareness campaigns, creating demonstration gardens, and removing invasive weeds from public spaces. These programs have proven to benefit everyone involved—the volunteers, the recipients of their services, their neighborhoods, and society.

Many more opportunities need to be created and designed so we can take full advantage of the growing numbers of older people who can, and want to, make a difference in their communities and our society. In our increasingly complex world, we cannot afford to overlook or ignore the contributions that older adults can make to building a better world for all.

Two key theoretical approaches to volunteering are those that emphasize explanations of volunteering and those that emphasize the process of volunteering [16]. Much of the literature has taken an explanatory theoretical approach, yielding important findings of the characteristics and motivations of volunteers.

### 1.2. Determinants of Volunteerism

Within the explanatory theoretical approach, the literature has identified numerous determinants of volunteering that support the resource theory of volunteering. The resource theory of volunteering posits that volunteerism depends on human capital (e.g., education, income, and health), social capital (e.g., social interaction), and cultural capital (e.g., religiosity) [17]. Many studies at the microlevel have shown that health and well-being are important human capital determinants of volunteerism among older adults [18–21]. More years of education, another form of human capital, has also been associated with increased volunteerism among older adults [18, 20].

At the mesolevel, a number of determinants of volunteerism that relate to social capital have been identified in the research on older adults. A study of older adults in Belgium found that altruism and weekly to daily contact with friends were both associated with increased volunteerism [19]. Another marker of social capital, a strong sense of community or neighborhood connectedness, a marker of social capital, has been associated with volunteerism among older adults [22, 23]. Neighborhood satisfaction has also been associated with volunteerism among older adults [22]. Rurality is associated with less volunteerism simply because these communities often lack opportunities [24]. Home ownership and more community amenities and services have also been associated with increased volunteerism among older adults [22]. Two cross-sectional studies found that safer neighborhood perception was associated with increased volunteerism among older adults [2, 20].

At the macrolevel, determinants of volunteering such as religiosity, a marker of cultural capital [17], have also consistently been associated with volunteerism among older adults [18, 19, 25]. The level of funding for programs such as those discussed previously is also a determinant of volunteerism among older adults. In recent years, there have been cuts to government-sponsored volunteer and training programs in the United States that serve older adults, which create a barrier to volunteerism at the societal level [26].

### 1.3. Perceived Neighborhood Safety and Volunteerism

This study takes another key theoretical approach to volunteering, a process-oriented approach that focuses on the role of the environment and examines volunteering across time. Neighborhood safety, a process-oriented and mesolevel environmental factor affecting volunteerism, has been studied less frequently, with a dearth of studies in the U.S. Perceived neighborhood safety can also be considered to be a proxy measure for social capital using the resource theory of aging [17] given that social capital depends on trust in others. Whether one perceives their neighborhood safety as good or poor is a marker of how much trust they have in the area in which they live. Previous cross-sectional studies have shown that perceptions of greater neighborhood safety are associated with increased levels of volunteerism among older adults [2, 20]. One study from Belgium examined the association from the other direction and found that older adults who volunteer are more likely to feel safe in their neighborhoods compared with older adults who do not volunteer [27]. Another study found that greater amounts of fear of violent and property crime are associated with reduced volunteerism [28].

More broadly, the literature shows that perceptions of an unsafe neighborhood are associated with a host of negative health outcomes while positive neighborhood safety perceptions are associated with health benefits. Poor perceived neighborhood safety is associated with more depression [29, 30], reductions in walking [31, 32], functional decline [33], worse health [29], and mortality [34, 35] among older adults. On the other hand, perceptions that neighborhoods are safe are associated with improved psychological well-being [36] and increased leisure-time physical activity [37] among older adults.

### 1.4. Gender and Race Differences in Perceived Safety and Volunteerism

There is an intersectional nature to the relationship between perceived safety and volunteerism among older adults. The fear-victimization paradox (alternately referred to as the sex-fear of crime paradox when discussing differences between males and females) is often cited when discussing higher rates of fear of victimization among older adults and specifically older women despite a lower prevalence of actual victimization among both older people and women [38, 39].

Women are more likely to perceive their neighborhoods as unsafe compared with men, and the effects of poor perceived neighborhood safety are typically, though not always, greater among women [28, 39–46]. Most studies also find that women volunteer at higher rates than men though a review of studies examining volunteerism across a number of countries found the differences to be sometimes small [47]. One study suggests that human capital, resources, and beliefs may account for observed differences in volunteerism between males and females [48]. In addition to formal volunteering, women are more likely to engage in informal volunteering; however, these informal efforts are often underestimated [13]. One study examining the effect of fear of crime on volunteerism and another examining the effect of perceived neighborhood safety on volunteerism found that the effect of fear of crime differed by gender, and the effect was larger among women [2, 28]. Given the previous research on gender differences in perceived neighborhood safety, volunteerism, and the association between the two, this relationship should be analyzed further in this research.

Less research has been conducted on the participation of members of racial or ethnic minority groups in volunteerism [49]. A scoping review of 55 years of published literature on older adults' civic participation found that only 1.7 percent of 429 articles addressed race or ethnicity [50]. Black Americans have a long tradition of civic engagement, and much of it focused on maintaining and developing local communities [51]. And while some estimates show a lower prevalence of volunteerism among minority older adults, these estimates often ignore the informal volunteerism contributions made by older, minority adults [52]. Research on perceived neighborhood characteristics also shows differences by race. Some research has shown that White respondents perceive more neighborhood disorder than Black respondents [53], yet research on older women has shown that older, Black women had significantly lower perceptions of safety compared with White women [54]. One cross-sectional study that examined the effect of perceived neighborhood safety on volunteerism stratified by race found that perceived neighborhood safety was only a significant determinant of volunteerism among White respondents and was not significant among non-Hispanic Asian, non-Hispanic Black, or Hispanic adults over the age of 50 years [20].

### 1.5. Hypotheses

We are interested in building upon previous work (mostly in non-US settings) to determine the extent to which a process-oriented environmental factor—perceived neighborhood safety—is associated with volunteerism among older Americans and how this effect differs by gender and race. The first hypothesis is that perceptions of better neighborhood safety will be associated with increased volunteerism among older adults after controlling for other model covariates. Given the research showing that perceived safety differs by gender [28, 39–46] and by race [20, 34, 55], the second and third hypotheses are that the effect of perceived neighborhood safety on volunteerism will differ by gender and by race.

## 2. Materials and Methods

### 2.1. Data Source and Sample

The data for these analyses come from the Health and Retirement Study (HRS), an ongoing longitudinal panel survey. Every two years since 1992, the HRS has interviewed approximately 20,000 Americans who are 50 years of age and older. Data are collected on a variety of topics including demographic characteristics, health behaviors and outcomes, employment status, and wealth and income. The HRS sample uses a multistage, stratified study design [56]. The analyses in this study used HRS data from 2008 to 2018 as 2008 was the first wave of data to assess the exposure and perceived neighborhood safety. Six waves of data were included because data are collected every two years. The sample was limited to respondents aged 60 years and older as the hypotheses pertain to older adults. The final sample size was 72,319.

### 2.2. Measures

The outcome variable, volunteerism, was assessed by asking, “Have you spent any time in the past 12 months doing volunteer work for religious, educational, health-related, or other charitable organizations?” Responses included yes/no. The exposure of interest, perceived neighborhood safety, was assessed by asking, “Would you say the safety of (your/that) neighborhood is excellent, very good, good, fair, or poor?” Original response categories were recoded to combine fair and poor into one category as there were small proportions of respondents in each of these two categories. Other covariates included in the measurement model were based on a literature review examining predictors of volunteerism and bivariate analysis with the outcome variable, volunteerism. Model covariates included gender (male/female), age (years), race (White, Black, and other), Hispanic ethnicity (Hispanic/not Hispanic), education (less than high school, GED, high school graduate, some college, and college and above), marital status (married/partnered, separated/divorced/widowed, and never married), employment status (works for pay, does not work for pay), total assets (dollars), attendance at religious services (never, at least once a year, two or three times per month, once a week, and more than once a week), depression (depressed, not depressed), and self-assessed health status (excellent, very good, good, fair, and poor). Total assets were used rather than income as retired older adults, who comprised 74.2 percent of our sample, may not have any income from a current job; however, they could have large amounts of assets. Assets are likely to be a more appropriate indicator of financial status and thus are a better proxy for actual neighborhood safety, allowing the model to better examine the effects of perceived safety while controlling for differences in neighborhoods.

### 2.3. Statistical Analyses

These data were analyzed using generalized estimation equations (GEE), which yield efficient estimates in the presence of correlated observations as is the case with repeated measures using panel data. These GEE models employed the binomial family and logit link to estimate volunteerism as a binary outcome. The QIC (quasi-information criterion) was used to determine that an independent correlation structure was the most appropriate for this model, which controls for the within-respondent correlation due to repeated measures across all waves of data [57]. In addition, cluster robust standard errors that employ the Huber-White sandwich estimator were used to correct for dependence among repeated observations, which allows estimates to be unbiased. An alpha of less than or equal to 0.05 was used to determine statistical significance. Given that we hypothesize that there may be differences in the effect of perceived neighborhood safety on volunteerism by gender and race, we tested perceived neighborhood safety by gender and by race interaction terms. Average marginal effects are presented as they are not affected by unobserved heterogeneity unrelated to the model's independent variables, which allows estimates to be compared across models [58]. All models are controlled for all listed covariates. All analyses were conducted using Stata 16.1.

## 3. Results

### 3.1. Demographic Characteristics

Approximately 58% of this sample was female with an average age of 73 years (see Table 1). A majority of respondents were White (77%), non-Hispanic (89%), and married or partnered (59%). A quarter of respondents were still working for pay (26%). Self-assessed health status of the sample was distributed across excellent (8%), very good (29%), good (34%), fair (22%), and poor (8%). A third of the sample volunteered in the past year (33%). A plurality of respondents regarded their neighborhood safety as excellent (34%) with an almost equal proportion assessing their neighborhood safety as very good (33%) and fewer reporting good (22%) or fair/poor (11%).

### 3.2. Overall Multivariate Models

Table 2 presents the results of the analyses for the entire sample. The probability of volunteering among respondents who rated their perceived neighborhood safety as excellent increased by three percentage points compared with respondents who rated their perceived neighborhood safety as fair/poor after controlling for all other model covariates (AME: 0.03, 95% CI: 0.02, 0.05). The probability of volunteering increased by two percentage points among those who rated their perceived neighborhood safety as very good compared with those who rated their perceived neighborhood safety as fair/poor after controlling for all other model covariates (AME: 0.02, 95% CI: 0.01, 0.04). There was no association between perceived neighborhood safety and volunteerism among respondents who rated their neighborhood safety as good.

Race/ethnicity was significantly associated with the probability of volunteering decreasing by three percentage points among Black participants (AME: -0.03, 95% CI: -0.04, -0.01), two percentage points among participants who reported another race (AME: -0.02, 95% CI: -0.05, -0.00), and nine percentage points among those identifying as Hispanic (AME: -0.09, 95% CI: -0.11, -0.08). Increasing levels of education were positively, significantly associated with the probability of volunteering. Compared with those who had less than a high school degree, those with a GED had an increased probability of volunteering by seven percentage points (AME: 0.07, 95% CI: 0.05, 0.10), those who were high school graduates had an increase in the probability of volunteering by nine percentage points (AME: 0.09, 95% CI: 0.07, 0.10), those with some college by 15 percentage points (AME: 0.15, 95% CI: 0.14, 0.17), and college graduates and higher by 26 percentage points (AME: 0.26, 95% CI: 0.24, 0.28). Respondents who were divorced, separated, or widowed had a decreased probability of volunteering by two percentage points (AME: -0.02, 95% CI: -0.03, -0.01) as did those who were never married (AME: -0.03, 95% CI: -0.06, -0.01). Those who were still working for pay were two percentage points more likely to volunteer compared with those who were not still working (AME: 0.02, 95% CI: 0.01, 0.03). Religious attendance had some of the largest effects on the probability of volunteering with those attending religious service more than once a week having a 52 percentage point increase in the probability of volunteering (AME: 0.52, 95% CI: 0.50, 0.53), those attending once per week having a 30 percentage point increase in the probability of volunteering (AME: 0.30, 95% CI: 0.29, 0.31), those attending two or three times per month having a 20 percentage point increase in the probability of volunteering (AME: 0.20, 95% CI: 0.19, 0.22), and those who attended once or more per year having a nine percentage point increase in the probability of volunteering (AME: 0.09, 95% CI: 0.08, 0.10) compared with those who did not attend religious services. Depression was associated with decreased probability of volunteering (AME: -0.03, 95% CI: -0.05, -0.02). Higher levels of self-assessed health status (SAHS) were positively associated with the probability of volunteering with excellent SAHS associated with an 18 percentage point increase in the probability of volunteering (AME: 0.18, 95% CI: 0.16, 0.20), very good SAHS associated with a 17 percentage point increase in the probability of volunteering (AME: 0.17, 95% CI: 0.15, 0.18), good SAHS associated with a 13 percentage point increase in the probability of volunteering (AME: 0.13, 95% CI: 0.11, 0.14), and fair SAHS associated with an eight percentage point increase in the probability of volunteering (AME: 0.08, 95% CI: 0.06, 0.09) compared with those reporting poor SAHS.

### 3.3. Multivariate Results by Gender

While the interaction terms for gender by perceived neighborhood safety were not significant, the literature has a number of papers showing differences in perceived neighborhood safety or fear of crime by gender [2, 41, 59–61]; thus, results were stratified for further examination by gender.  Table 3 presents the results stratified by gender. Results among males were only significant for those who perceived their neighborhood safety to be excellent. Among males rating their neighborhood safety as excellent, the probability of volunteering increased by four percentage points after controlling for all other covariates (AME: 0.04, 95% CI: 0.02, 0.07). Among females, the probability of volunteering increased by three percentage points among those who perceived their neighborhood safety to be excellent (AME: 0.03, 95% CI: 0.01, 0.05) and by two percentage points among those who perceived their neighborhood safety to be very good (AME: 0.02, 95% CI: 0.00, 0.04).

Identifying as another race was associated with a four percentage point decrease in the probability of volunteering (AME: -0.04, 95% CI: -0.07, -0.01) among males. Black race was associated with a four percentage point decrease in the probability of volunteering among females (AME: -0.04, 95% CI: -0.05, -0.02). Education level was positively correlated with increasing probabilities of volunteering among both males and females. Being divorced, separated, or widowed was associated with a five percentage point decrease in the probability of volunteering among males (AME: -0.05, 95% CI: -0.06, -0.03), and never married was associated with a decrease of six percentage points among males (AME: -0.06, 95% CI: -0.10, -0.02) compared with those who were married. Marital status was not associated with volunteering among females. Both males and females had similar positive correlations between increasing levels of religious attendance and increasing probabilities of volunteering with the effects being slightly stronger for each level of religious attendance among males.

### 3.4. Multivariate Results by Race

Table 4 presents the results stratified by race. The interaction term for race by perceived neighborhood safety was significant for many of the race by perceived neighborhood safety combinations, which aligns with much of the literature on perceived neighborhood safety [34]. Among White respondents, those who rated their neighborhood safety as excellent had an increased probability of volunteering by five percentage points (AME: 0.05, 95% CI: 0.03, 0.07) while those reporting very good perceived neighborhood safety had an increased probability of volunteering of four percentage points (AME: 0.04, 95% CI: 0.02, 0.06) compared to those who rated their neighborhood safety as fair or poor after controlling for all other model covariates. Results for Black respondents and those who described their race as “other” were not significant for any of the levels of perceived neighborhood safety. There was no association between perceived neighborhood safety and volunteerism for respondents who were not White.

Those who identify as Hispanic and White were 11 percentage points less likely to volunteer (AME: -0.11, 95% CI: -0.13, -0.09) while those identifying as Hispanic and another race were four percentage points less likely to volunteer (AME: -0.04, 95% CI: -0.08, -0.00) compared with those who did not identify as Hispanic. Increasing levels of education were associated with increased probabilities of volunteering among all races. Being divorced, separated, or widowed was associated with a two percentage point decrease in the probability of volunteering among White respondents (AME: -0.02, 95% CI: -0.03, -0.01) and a three percentage point decrease among Black respondents (AME: -0.03, 95% CI: -0.06, -0.01). Having never been married was associated with a decrease of five percentage points among Black respondents (AME: -0.05, 95% CI: -0.09, -0.00) compared with those who were married. Marital status was not associated with volunteering among those identifying as another race. Working for pay was associated with increases in volunteering, with larger and more significant increases among Black respondents (AME: 0.05, 95% CI: 0.02, 0.07) and those identifying as another race (AME: 0.05, 95% CI: 0.02, 0.09) and somewhat smaller effects among White respondents (AME: 0.01, 95% CI: 0.00, 0.02). Religious attendance was similarly positively associated with the probability of volunteering across all three racial groups. Depression was associated with a four percentage point reduction in the probability of volunteering among White respondents (AME: -0.04, 95% CI: -0.06, -0.03) and those reporting another race (AME: -0.04, 95% CI: -0.08, -0.01), though depression was not associated with volunteering among Black respondents. Self-assessed health status was similarly positively correlated with increasing probabilities of volunteering among White and Black respondents. However, three of the four categories of self-assessed health status were not significantly associated with the probability of volunteering. Only very good SAHS was significantly associated with an eight percentage point increase in the probability of volunteering among those reporting another race compared with those reporting poor health (AME: 0.08, 95% CI: 0.02, 0.14).

## 4. Discussion

The results of this study provide further support for the association between perceived neighborhood safety and volunteerism; however, this association differs by demographic characteristics among older adults. The association between perceived neighborhood safety and volunteerism was slightly stronger and more significant among males though only among those who perceived their neighborhood safety to be excellent. The association between perceived neighborhood safety and volunteerism differed greatly by race. While results were strong and significant for White respondents, with better perceived neighborhood safety being associated with more volunteering, there was no association between perceived neighborhood safety and volunteerism among Black respondents or those who reported “other” as their race.

These findings are consistent with previous literature examining the resource theory of volunteering [17, 62] but also add to the existing literature on this topic. Previous studies have shown an association between volunteerism and other forms of social capital that foster volunteerism such as civic engagement and neighborhood factors such as social cohesion, social capital, and neighborhood connectedness [20, 22, 63]. In addition, previous work specifically examining this relationship cross-sectionally also found that perceptions of safer neighborhoods, which could be a proxy for social capital in the resource theory of aging, were associated with volunteerism among older adults [2, 20]. Confirming this relationship using longitudinal data strengthens these findings. However, the results are not entirely consistent with previous literature. The previous study examining perceived neighborhood safety and volunteerism found stronger and more significant associations among females [2]. It is possible that the previous study, which only used data from one year, found an effect specifically associated with that particular year of study, which was not consistent with the overall results across many years of study. This provides additional support for the value of more longitudinal research on these topics. On the other hand, another study examining the effect of perceived neighborhood safety on mortality did find a stronger and more significant association among males [35], so there is some support in other areas that perceived neighborhood safety may have a stronger effect among males.

An examination of cross-sectional data from California found differences in the association between perceived neighborhood safety by race [20]. That study's results also showed that White respondents who reported living in safer neighborhoods had increased odds of volunteering though there was no significant effect among non-Hispanic Asian, non-Hispanic Black, or Hispanic respondents [20]. Furthermore, other research has shown stronger effects of perceived neighborhood safety on health outcomes, including mortality, among White populations [34]. This paper builds upon that understanding using longitudinal data from across the US that also shows a stronger, more significant effect of perceived neighborhood safety on volunteerism among White populations.

### 4.1. Strengths and Limitations

This study has a number of strengths. This research was conducted on a large sample of older adults in the US. One of the biggest strengths is the ability to analyze these data longitudinally using robust standard errors and accounting for the intrarespondent correlation so that unbiased, efficient estimates can be produced. The HRS dataset includes many covariates that can be used to control for a number of known potential confounders in the relationship between perceived neighborhood safety and volunteerism. This allows for a better understanding of the actual effect of perceived neighborhood safety on volunteerism. Another benefit of the HRS data is being able to control for assets rather than income because assets are a better indicator of financial resources among older adults and can also be considered to be an adequate proxy for actual neighborhood safety.

A number of limitations to this study should be noted. Measurement error is possible with certain variables including the primary exposure. Perceived neighborhood safety has been measured in myriad ways throughout the literature. The measure taken from the HRS dataset has not been validated, so it is possible that there is measurement error in this question. In addition, volunteerism was assessed using a dichotomous question. Perhaps a more nuanced assessment of the amount respondents volunteered would allow for additional analysis of the data. While the HRS is a comprehensive dataset, the measurement model did not include every conceptual variable that is likely to determine volunteerism. In addition, there are no contextual variables, such as crime rates, available in this dataset that could be important to examine as measures of actual neighborhood safety. There is also concern that the concept of volunteerism differs among racially diverse respondents and females [13, 52]. Older adults of color and older women likely engage in more informal volunteering, which may not be captured by the question used in the HRS data leading to an underestimate of volunteerism among these groups. While the HRS does ask respondents about providing unpaid help to friends, neighbors, or relatives, this is a limited assessment of informal volunteerism and is also likely to underestimate these acts among female and minority subgroups.

### 4.2. Implications

From a process-oriented theoretical perspective, these analyses provide support for the hypothesis that an environmental factor—in this study, better perceived neighborhood safety—is associated with increased volunteerism among older adults and that there are slight gender and larger race differences in this association. Understanding the subgroup differences will allow for more targeted interventions in an effort to ultimately improve volunteerism among older adults, which has benefits for the individual, the social environment, and society. Many more opportunities need to be created and designed so we can take full advantage of the growing numbers of older people who can, and want to, make a difference in their communities and our society. In an increasingly complex world, we cannot afford to overlook or ignore the contributions that older adults can make and the resources they can bring to building a better world for all.

Research should endeavor to better understand the fear-victimization paradox. If older, White adults are disproportionately afraid, efforts should be made to address these fears so that this deterrent to volunteerism can be minimized. Research should also determine why older, White adults reduce their volunteerism when they perceive their neighborhoods to be less safe. Data should be collected that include more environmental-level variables in an effort to develop more robust measurement models. More perceived area-level variables should be added to datasets including social cohesion, and datasets with contextual information should be analyzed so that the effects of area-level variables such as crime rates and poverty can be added to models that largely focus on individual-level attributes and primarily perceived, but not actual, neighborhood-level characteristics.

Volunteerism can be good for individuals, the environment in which they live, and society as a whole. Programs and policies that lead to improved volunteerism can benefit all three. In the US, there have been structural barriers making volunteerism more difficult, including funding cuts to government-sponsored volunteer and training programs for older adults such as Senior Corps [26]. With reduced funding, there may be fewer opportunities that feel acceptable specifically for older adults who perceive their neighborhoods to be less safe. These barriers must be acknowledged and addressed with increased governmental funding to enable societies in which volunteer opportunities are readily available for all older adults. Communities can develop their own volunteer opportunities for older adults. Community and capacity building could involve creating opportunities for older adults to engage in activities that can be carried out at home or during the day, which may prevent the suppression of volunteerism associated with worse perceived neighborhood safety and could address some of the macrolevel suppressants identified by Gonzales et al. [26].

There may be overlap between those who perceive their neighborhoods as less safe and those who lack access to volunteer opportunities. During the recent COVID-19 pandemic, when shelter-in-place orders initially prevented many from volunteering, it was important for communities to develop virtual volunteering opportunities for older adults [64]. While often less preferable due to concerns about social isolation, virtual volunteer opportunities that have become more widespread during this pandemic could also be used to engage older adults who perceive their neighborhoods to be less safe. Previous research has shown that social connectedness and integration are key determinants of volunteerism [19, 65]. Leveraging social connectedness in a virtual world could engage older adults who perceive their neighborhoods to be unsafe. The prevalence of virtual volunteering is higher among males, and given the larger and more significant effect of perceived neighborhood safety on volunteerism among males, this may be a successful avenue of intervention at the mesolevel.

On the microlevel, programs should be developed to improve perceived neighborhood safety, thereby improving social capital and adding to the resources available to older adults both generally and specifically while volunteering. Research has examined older adults' motivation for volunteering, which could also be important in developing programs to encourage volunteerism [66, 67]. One study found that among older adults who were still working, motivations to volunteer may be driven by opportunities to improve careers [67]. Developing a better understanding of the motivations to volunteer among older adults who perceive their neighborhoods as less safe is an important step in developing programs to fit their needs. One study found that lower amounts of human and social capital, of which poorer neighborhood safety could be considered, were associated with volunteering as a method of enhancing self-esteem and avoidance of thinking about problems and for social benefits [66]. This understanding could help organizations develop opportunities for older adults who perceive their neighborhood to be less safe, as well. Volunteerism is one aspect of age-friendly communities that encourages aging in place [68]. Thus, developing micro-, meso-, and macrolevel interventions to address perceived neighborhood safety could increase human, social, and cultural capital that could, in turn, benefit older adults, their communities, and society.

## 5. Conclusion

This longitudinal assessment of the effect of perceived neighborhood safety on volunteerism has confirmed this association and elucidated gender and race differences in the association between the two. Previous literature has shown the numerous individual, community, and societal benefits of volunteering. Creating volunteer opportunities for older adults that address concerns about neighborhood safety will allow for increased volunteerism as well as its associated benefits.

## Figures and Tables

**Table 1:** Sample characteristics.

Variable	*N* (%)	Mean (SD)
Volunteered (past year)	25,729 (33.2)	
Perceived neighborhood safety		
Excellent	26,276 (33.9)	
Very good	25,755 (33.2)	
Good	17,254 (22.2)	
Fair/poor	8,300 (10.7)	
Gender		
Female	44,947 (57.9)	
Male	32,638 (42.1)	
Age		72.5 (8.66)
Race		
White	59,573 (76.9)	
Black	13,095 (93.8)	
Other race	4,821 (6.2)	
Hispanic ethnicity	8,905 (11.5)	
Education		
Less than HS degree	15,662 (20.2)	
GED	3,602 (4.6)	
HS degree	23,216 (29.9)	
Some college	17,977 (23.2)	
College grad and higher	17,108 (22.1)	
Marital status		
Married	46,030 (59.4)	
Divorced/separated/widowed	28,698 (37.0)	
Never married	2,796 (3.6)	
Working for pay	19,999 (25.8)	
Total assets ($)		503,056 (1,585,032)
Religiosity		
>once/week	11,649 (15.1)	
Once/week	20,782 (26.9)	
Two/three times/month	9,422 (12.2)	
Once or more/year	14,676 (19.0)	
Not at all	20,689 (26.8)	
Depressed (past year)	8,983 (12.3)	
Self-assessed health status		
Excellent	6,004 (7.8)	
Very good	22,082 (28.5)	
Good	25,940 (33.5)	
Fair	17,087 (22.0)	
Poor	6,407 (8.3)	

**Table 2:** Multivariate logistic regression models estimating the average marginal effect of each predictor on volunteerism among older adults (*n* = 72,319)^∗^.

Variable	Average marginal effect	95% confidence interval	*p* value
Perceived neighborhood safety (Ref: fair/poor)			
Excellent	**0.03**	(0.02, 0.05)	<0.0001
Very good	**0.02**	(0.01, 0.04)	0.01
Good	-0.00	(-0.02, 0.01)	0.76
Female gender	0.01	(-0.00, 0.02)	0.27
Age	**-0.00**	(-0.00, -0.00)	<0.0001
Race (Ref: White)			
Black	**-0.03**	(-0.04, -0.01)	<0.0001
Other	**-0.02**	(-0.05, -0.00)	0.03
Hispanic ethnicity	**-0.09**	(-0.11, -0.08)	<0.0001
Education (Ref: less than HS)			
GED	**0.07**	(0.05, 0.10)	<0.0001
High school graduate	**0.09**	(0.07, 0.10)	<0.0001
Some college	**0.15**	(0.14, 0.17)	<0.0001
College grad and higher	**0.26**	(0.24, 0.28)	<0.0001
Marital status (Ref: married)			
Divorced/separated/widowed	**-0.02**	(-0.03, -0.01)	<0.0001
Never married	**-0.03**	(-0.06, -0.01)	0.01
Working for pay	**0.02**	(0.01, 0.03)	<0.0001
Total assets ($)	1.29**e** − 0.09	(1.00, 1.00)	<0.0001
Religious attendance (Ref: not at all)			
>once/week	**0.52**	(0.50, 0.53)	<0.0001
Once/week	**0.30**	(0.29, 0.31)	<0.0001
Two/three times/month	**0.20**	(0.19, 0.22)	<0.0001
Once or more/year	**0.09**	(0.08, 0.10)	<0.0001
Depressed	**-0.03**	(-0.05, -0.02)	<0.0001
Self-assessed health status (Ref: poor)			
Excellent	**0.18**	(0.16, 0.20)	<0.0001
Very good	**0.17**	(0.15, 0.18)	<0.0001
Good	**0.13**	(0.11, 0.14)	<0.0001
Fair	**0.08**	(0.06, 0.09)	<0.0001

^∗^Results in bold indicate statistical significance at the alpha = 0.05 level.

**Table 3:** Multivariate logistic regression models estimating the average marginal effect of each predictor on volunteerism among older adults, by gender^∗^.

Variable	Males			Females		
	Average marginal effect	95% confidence interval	*p* value	Average marginal effect	95% confidence interval	*p* value
Perceived neighborhood safety (Ref: fair/poor)						
Excellent	**0.04**	(0.02, 0.07)	<0.0001	**0.03**	(0.01, 0.05)	0.01
Very good	0.02	(-0.00, 0.05)	0.07	**0.02**	(0.00, 0.04)	0.05
Good	0.00	(-0.02, 0.03)	0.76	-0.01	(-0.03, 0.01)	0.46
Age	**-0.01**	(-0.01, -0.00)	<0.0001	**-0.00**	(-0.00, -0.00)	<0.0001
Race (Ref: White)						
Black	-0.01	(-0.03, 0.01)	0.36	**-0.04**	(-0.05, -0.02)	<0.0001
Other	**-0.04**	(-0.07, -0.01)	0.02	-0.02	(-0.04, 0.01)	0.31
Hispanic ethnicity	**-0.09**	(-0.12, -0.06)	<0.0001	**-0.09**	(-0.12, -0.07)	<0.0001
Education (Ref: less than HS)						
GED	**0.09**	(0.05, 0.12)	<0.0001	**0.06**	(0.03, 0.09)	<0.0001
High school graduate	**0.09**	(0.06, 0.11)	<0.0001	**0.09**	(0.07, 0.11)	<0.0001
Some college	**0.13**	(0.11, 0.16)	<0.0001	**0.17**	(0.15, 0.19)	<0.0001
College grad and higher	**0.23**	(0.20, 0.25)	<0.0001	**0.29**	(0.27, 0.32)	<0.0001
Marital status (Ref: married)						
Divorced/separated/widowed	**-0.05**	(-0.06, -0.03)	<0.0001	-0.01	(-0.02, 0.01)	0.44
Never married	**-0.06**	(-0.10, -0.02)	0.007	-0.02	(-0.05, 0.02)	0.34
Working for pay	**0.03**	(0.02, 0.05)	<0.0001	0.01	(-0.00, 0.03)	0.11
Total assets ($)	1.11 **e** − 08	(5.82*e* − 09, 1.63*e* − 08)	<0.0001	1.60**e** − 08	(8.55*e* − 09, 2.35*e* − 08)	<0.0001
Religious attendance (Ref: not at all)						
>once/week	**0.57**	(0.54, 0.59)	<0.0001	**0.49**	(0.47, 0.51)	<0.0001
Once/week	**0.33**	(0.31, 0.35)	<0.0001	**0.28**	(0.26, 0.29)	<0.0001
Two/three times/month	**0.22**	(0.20, 0.24)	<0.0001	**0.19**	(0.17, 0.20)	<0.0001
Once or more/year	**0.22**	(0.20, 0.24)	<0.0001	**0.08**	(0.07, 0.10)	<0.0001
Depressed	**-0.02**	(-0.04, 0.00)	0.05	**-0.04**	(-0.05, -0.02)	<0.0001
Self-assessed health status (Ref: poor)						
Excellent	**0.16**	(0.12, 0.19)	<0.0001	**0.19**	(0.16, 0.22)	<0.0001
Very good	**0.14**	(0.11, 0.17)	<0.0001	**0.19**	(0.16, 0.21)	<0.0001
Good	**0.12**	(0.09, 0.14)	<0.0001	**0.13**	(0.11, 0.16)	<0.0001
Fair	**0.09**	(0.06, 0.11)	<0.0001	**0.07**	(0.05, 0.09)	<0.0001

^∗^Results in bold indicate statistical significance at the alpha = 0.05 level.

**Table 4:** Multivariate logistic regression models estimating the average marginal effect of each predictor on volunteerism among older adults, by race^∗^.

Variable	White			Black			Other		
	Average marginal effect	95% confidence interval	*p* value	Average marginal effect	95% confidence interval	*p* value	Average marginal effect	95% confidence interval	*p* value
Perceived neighborhood safety (Ref: fair/poor)									
Excellent	**0.05**	(0.03, 0.07)	<0.0001	-0.01	(-0.04, 0.03)	0.74	0.02	(-0.03, 0.06)	0.46
Very good	**0.04**	(0.02, 0.06)	0.001	-0.00	(-0.03, 0.03)	0.94	-0.00	(-0.04, 0.04)	0.94
Good	0.01	(-0.01, 0.03)	0.41	-0.00	(-0.03, 0.02)	0.79	-0.02	(-0.05, 0.02)	0.40
Female gender	**0.01**	(-0.00, 0.02)	0.05	**-0.03**	(-0.06, -0.01)	0.01	0.00	(-0.03, 0.04)	0.86
Age	**-0.00**	(-0.01, -0.00)	<0.0001	**-0.00**	(-0.00, -0.00)	<0.0001	**-0.00**	(-0.01, -0.00)	0.003
Hispanic ethnicity	**-0.11**	(-0.13, -0.09)	<0.0001	-0.01	(-0.10, 0.09)	0.91	**-0.04**	(-0.08, -0.00)	0.04
Education (Ref: less than HS)									
GED	**0.06**	(0.03, 0.09)	<0.0001	**0.07**	(0.02, 0.12)	0.003	**0.15**	(0.08, 0.23)	<0.0001
High school graduate	**0.09**	(0.07, 0.11)	<0.0001	**0.07**	(0.04, 0.10)	<0.0001	**0.08**	(0.04, 0.13)	<0.0001
Some college	**0.15**	(0.13, 0.17)	<0.0001	**0.16**	(0.12, 0.19)	<0.0001	**0.18**	(0.13, 0.23)	<0.0001
College grad and higher	**0.26**	(0.24, 0.28)	<0.0001	**0.25**	(0.21, 0.29)	<0.0001	**0.23**	(0.17, 0.29)	<0.0001
Marital status (Ref: married)									
Divorced/separated/widowed	**-0.02**	(-0.03, -0.01)	0.003	**-0.03**	(-0.06, -0.01)	0.01	0.03	(-0.00, 0.07)	0.06
Never married	-0.03	(-0.06, 0.01)	0.15	**-0.05**	(-0.09, -0.00)	0.03	-0.03	(-0.09, 0.04)	0.42
Working for pay	**0.01**	(0.00, 0.02)	0.05	**0.05**	(0.02, 0.07)	<0.0001	**0.05**	(0.02, 0.09)	0.002
Total assets ($)	1.14 **e** − 08	(7.09*e* − 09, 1.56*e* − 08)	<0.0001	8.75 **e** − 08	(4.86*e* − 08, 1.26*e* − 07)	<0.0001	2.62**e** − 08	(8.21*e* − 09, 4.43*e* − 08)	0.004
Religious attendance (Ref: not at all)									
>once/week	**0.52**	(0.50, 0.54)	<0.0001	**0.53**	(0.50, 0.56)	<0.0001	**0.47**	(0.42, 0.53)	<0.0001
Once/week	**0.32**	(0.30, 0.33)	<0.0001	**0.29**	(0.26, 0.31)	<0.0001	**0.17**	(0.13, 0.21)	<0.0001
Two/three times/month	**0.21**	(0.19, 0.22)	<0.0001	**0.21**	(0.18, 0.24)	<0.0001	**0.14**	(0.10, 0.19)	<0.0001
Once or more/year	**0.09**	(0.08, 0.11)	<0.0001	**0.08**	(0.06, 0.11)	<0.0001	**0.05**	(0.02, 0.08)	0.003
Depressed	**-0.04**	(-0.06, -0.03)	<0.0001	0.00	(-0.02, 0.03)	0.91	**-0.04**	(-0.08, -0.01)	0.02
Self-assessed health status (Ref: poor)									
Excellent	**0.19**	(0.17, 0.22)	<0.0001	**0.13**	(0.07, 0.18)	<0.0001	0.03	(-0.04, 0.10)	0.40
Very good	**0.18**	(0.16, 0.20)	<0.0001	**0.15**	(0.11, 0.19)	<0.0001	**0.08**	(0.02, 0.14)	0.006
Good	**0.14**	(0.12, 0.16)	<0.0001	**0.12**	(0.09, 0.16)	<0.0001	0.02	(-0.03, 0.07)	0.41
Fair	**0.08**	(0.06, 0.10)	<0.0001	**0.09**	(0.05, 0.12)	<0.0001	-0.00	(-0.05, 0.04)	0.86

^∗^Results in bold indicate statistical significance at the alpha = 0.05 level.

## Data Availability

The Health and Retirement Study data are freely available to all researchers.

## References

[1] AmeriCorps Older adults (age 65+). https://www.nationalservice.gov/vcla/demographic/older-adults-age-65.

[2] Grinshteyn E. G., Sugar J. A. (2020). Perceived neighbourhood safety and volunteerism among older adults. *Ageing and Society*.

[3] Huo M., Miller L. M. S., Kim K., Liu S. (2021). Volunteering, self-perceptions of aging, and mental health in later life. *The Gerontologist*.

[4] Jongenelis M. I., Jackson B., Newton R. U., Pettigrew S. (2021). Longitudinal associations between formal volunteering and well-being among retired older people: follow-up results from a randomized controlled trial. *Aging & Mental Health*.

[5] Lawton R. N., Gramatki I., Watt W., Fujiwara D. (2021). Does volunteering make us happier, or are happier people more likely to volunteer? Addressing the problem of reverse causality when estimating the wellbeing impacts of volunteering. *Journal of Happiness Studies*.

[6] Han S. H., Kim K., Burr J. A. (2018). Stress-buffering effects of volunteering on salivary cortisol: results from a daily diary study. *Social Science & Medicine*.

[7] Kim E. S., Whillans A. V., Lee M. T., Chen Y., VanderWeele T. J. (2020). Volunteering and Subsequent Health and Well-Being in Older Adults: An Outcome- Wide Longitudinal Approach. *American Journal of Preventive Medicine*.

[8] Morrow-Howell N., Hong S.-I., Tang F. (2009). Who benefits from volunteering? Variations in perceived benefits. *The Gerontologist*.

[9] Miller K. D., Schleien S. J., Rider C., Hall C., Roche M., Worsley J. (2002). Inclusive volunteering: benefits to participants and community. *Therapeutic Recreation Journal*.

[10] Madsen W., Ambrens M., Ohl M. (2019). Enhancing resilience in community-dwelling older adults: a rapid review of the evidence and implications for public health practitioners. *Frontiers in Public Health*.

[11] Ohmer M. L. (2007). Citizen participation in neighborhood organizations and its relationship to volunteers’ self- and collective efficacy and sense of community. *Social Work Research*.

[12] Woolley F. (2003). Social cohesion and voluntary activity: making connections. *The Economic Implications of Social Cohesion*.

[13] Warburton J., McLaughlin D. (2006). Doing it from your heart: the role of older women as informal volunteers. *Journal of Women & Aging*.

[14] Carr D. C., Fried L. P., Rowe J. W. (2015). Productivity & engagement in an aging America: the role of volunteerism. *Daedalus*.

[15] Pillemer K., Wells N. M., Meador R. H., Schultz L., Henderson C. R., Cope M. T. (2016). Engaging older adults in environmental volunteerism: the retirees in service to the environment program. *The Gerontologist*.

[16] Hustinx L., Cnaan R. A., Handy F. (2010). navigating theories of volunteering: a hybrid map for a complex phenomenon. *Journal for the Theory of Social Behaviour*.

[17] Wilson J., Musick M. (1997). Who cares? Toward an integrated theory of volunteer work. *American Sociological Review*.

[18] Choi L. H. (2003). Factors Affecting Volunteerism among Older Adults. *Journal of Applied Gerontology*.

[19] Dury S., De Donder L., De Witte N., Buffel T., Jacquet W., Verté D. (2015). To volunteer or Not. *Nonprofit and Voluntary Sector Quarterly*.

[20] Johnson K. J., Lee S. H. (2017). Factors associated with volunteering among racial/ethnic groups: findings from the California Health Interview Survey. *Research on Aging*.

[21] Wei Y., Donthu N., Bernhardt K. L. (2012). Volunteerism of older adults in the United States. *International Review on Public and Nonprofit Marketing*.

[22] Dury S., Willems J., De Witte N., De Donder L., Buffel T., Verté D. (2016). Municipality and neighborhood influences on volunteering in later life. *Journal of Applied Gerontology*.

[23] Okun M. A., Michel J. (2006). Sense of Community and Being a Volunteer Among the Young-Old. *Journal of Applied Gerontology*.

[24] Torgerson M., Edwards M. E. (2013). Demographic Determinants of Perceived Barriers to Community Involvement: Examining Rural/Urban Differences. *Nonprofit and Voluntary Sector Quarterly*.

[25] Musick M. A., Wilson J., Bynum W. B. (2000). Race and formal volunteering: the differential effects of class and religion. *Social Forces*.

[26] Gonzales E., Matz-Costa C., Morrow-Howell N. (2015). Increasing opportunities for the productive engagement of older adults: a response to population aging. *The Gerontologist*.

[27] De Donder L., De Witte N., Buffel T., Dury S., Verte D. (2012). Social capital and feelings of unsafety in later Life. *Research on Aging*.

[28] Britto S., Van Slyke D. M., Francis T. I. (2011). The role of fear of crime in donating and Volunteering. *Criminal Justice Review*.

[29] Roh S., Jang Y., Chiriboga D. A., Kwag K. H., Cho S., Bernstein K. (2011). Perceived neighborhood environment affecting physical and mental health: a study with Korean American older adults in New York City. *Journal of Immigrant and Minority Health*.

[30] Wilson-Genderson M., Pruchno R. (2013). Effects of neighborhood violence and perceptions of neighborhood safety on depressive symptoms of older adults. *Social Science & Medicine*.

[31] Loukaitou-Sideris A., Eck J. E. (2007). Crime prevention and active living. *American Journal of Health Promotion*.

[32] Van Cauwenberg J., De Donder L., Clarys P. (2014). Relationships between the perceived neighborhood social environment and walking for transportation among older adults. *Social Science & Medicine*.

[33] Sun V. K., Stijacic Cenzer I., Kao H., Ahalt C., Williams B. A. (2012). How safe is your neighborhood? Perceived neighborhood safety and functional decline in older adults. *Journal of General Internal Medicine*.

[34] Assari S. (2017). Perceived neighborhood safety better predicts risk of mortality for whites than blacks. *Journal of Racial and Ethnic Health Disparities*.

[35] Grinshteyn E., Muennig P., Pabayo R. (2019). Using the general social survey – National Death Index cohort to study the relationship between neighbourhood fear and mortality in the USA. *BMJ Open*.

[36] Choi Y. J., Matz-Costa C. (2018). Perceived neighborhood safety, social cohesion, and psychological health of older adults. *The Gerontologist*.

[37] Tucker-Seeley R. D., Subramanian S. V., Li Y., Sorensen G. (2009). Neighborhood safety, socioeconomic status, and physical activity in older adults. *American Journal of Preventive Medicine*.

[38] Britto S., Stoddart D., Bernat F. P., Frailing K. (2019). Fear, gender, and victimization. *The Encyclopedia of Women and Crime*.

[39] LaGrange R. L., Ferraro K. F. (1989). Assessing age and gender differences in perceived risk and fear of crime. *Criminology*.

[40] Assari S., Caldwell C. H., Zimmerman M. A. (2015). Perceived neighborhood safety during adolescence predicts subsequent deterioration of subjective health two decades later; gender differences in a racially-diverse sample. *International Journal of Preventive Medicine*.

[41] Ferraro K. F. (1995). *Fear of Crime: Interpreting Victimization Risk*.

[42] Ferraro K. F., LaGrange R. L. (1988). Are older people afraid of crime?. *Journal of Aging Studies*.

[43] Ferraro K. F., LaGrange R. L. (1992). Are older people most afraid of crime? Reconsidering age differences in fear of victimization. *Journal of Gerontology*.

[44] LaGrange R. L., Ferraro K. F. (1987). The elderly’s fear of crime: a critical examination of the research. *Research on Aging*.

[45] LaGrange R. L., Ferraro K. F. (2016). *The elderly’s fear of crime*.. *The Fear of Crime*.

[46] van Dyck D., Cerin E., de Bourdeaudhuij I. (2015). Moderating effects of age, gender and education on the associations of perceived neighborhood environment attributes with accelerometer-based physical activity: the IPEN adult study. *Health & Place*.

[47] Einolf C. J. (2011). Gender differences in the correlates of volunteering and charitable giving. *Nonprofit and Voluntary Sector Quarterly*.

[48] Wilson J. (2000). Volunteering. *Annual Review of Sociology*.

[49] Tang F., Copeland V. C., Wexler S. (2012). Racial Differences in Volunteer Engagement by Older Adults: An Empowerment Perspective. *Social Work Research*.

[50] Serrat R., Scharf T., Villar F., Gómez C. (2019). Fifty-five years of research into older people’s civic participation: recent trends, future directions. *The Gerontologist*.

[51] Chen H., Morrow-Howell N. (2015). Antecedents and outcomes of older adults’ motivations to volunteer with Experience Corps®. *Research in Human Development*.

[52] Martinez I. L., Crooks D., Kim K. S., Tanner E. (2011). Invisible civic engagement among older adults: valuing the contributions of informal volunteering. *Journal of Cross-Cultural Gerontology*.

[53] Sullivan D. M., Bachmeier J. D. (2012). Racial differences in perceived disorder in three gentrifying neighborhoods. *Advances in Applied Sociology*.

[54] Li W., Procter-Gray E., Youssef A. G. (2017). Racial differences in neighborhood perceptions and their influences on physical activity among urban older women. *AIMS Public Health*.

[55] Clark C. R., Kawachi I., Ryan L., Ertel K., Fay M. E., Berkman L. F. (2009). Perceived neighborhood safety and incident mobility disability among elders: the hazards of poverty. *BMC Public Health*.

[56] Ofstedal M. B., Weir D. R., Chen K.-T., Wagner J. (2011). *Updates to HRS sample weights*.

[57] Stata Xtgee—Fit Population-Averaged Panel-Data Models by Using GEE. Stata Manuals.. https://www.stata.com/manuals/xtxtgee.pdf.

[58] Mood C. (2010). Logistic regression: why we cannot do what we think we can do, and what we can do about it. *European Sociological Review*.

[59] Collins R. E. (2016). Addressing the inconsistencies in fear of crime research: a meta-analytic review. *Journal of Criminal Justice*.

[60] Hale C. (1996). Fear of crime: a review of the literature. *International Review of Victimology*.

[61] Snedker K. A. (2015). Neighborhood conditions and fear of crime. *Crime & Delinquency*.

[62] Principi A., Galenkamp H., Papa R. (2016). Do predictors of volunteering in older age differ by health status?. *European Journal of Ageing*.

[63] Pearce S., Kristjansson E. (2019). Physical and social perceptions of the neighbourhood and youth volunteerism: Canada’s capital region. *Canadian Journal of Nonprofit and Social Economy Research*.

[64] Lachance E. L. (2021). COVID-19 and its impact on volunteering: moving towards virtual volunteering. *Leisure Sciences*.

[65] Dury S., Brosens D., Smetcoren A.-S. (2020). Pathways to late-life volunteering: a focus on social connectedness. *Nonprofit and Voluntary Sector Quarterly*.

[66] Principi A., Schippers J., Naegele G., Di Rosa M., Lamura G. (2016). Understanding the link between older volunteers’ resources and motivation to volunteer. *Educational Gerontology*.

[67] Principi A., Warburton J., Schippers J., Rosa M. D. (2013). The role of work status on European older volunteers’ motivation. *Research on Aging*.

[68] Wiersma E. C., Koster R. (2013). Vulnerability, volunteerism, and age-friendly communities: placing rural northern communities into context. *Journal of Rural and Community Development*.

